# Editorial: Insights in plant biotechnology: 2021

**DOI:** 10.3389/fpls.2023.1147930

**Published:** 2023-01-30

**Authors:** James R. Lloyd, Ralf Wilhelm, Manoj K. Sharma, Jens Kossmann, Peng Zhang

**Affiliations:** ^1^ Institute for Plant Biotechnology, Department of Genetics, Stellenbosch University, Stellenbosch, South Africa; ^2^ Institute for Biosafety in Plant Biotechnology, Julius Kühn-Institute - Federal Research Centre for Cultivated Plants, Quedlinburg, Germany; ^3^ Crop Genetics and Informatics Group, School of Biotechnology, Jawaharlal Nehru University, New Delhi, India; ^4^ CAS Center for Excellence in Molecular Plant Sciences, Chinese Academy of Sciences, Shanghai, China

**Keywords:** abiotic and biotic stresses, plant biotechnology and breeding, pharmaceuticals, recombinant protein, genome edited crops

The Plant Biotechnology section at Frontiers in Plant Science mainly publishes applied studies examining how plants can be improved using modern genetic techniques (Lloyd and Kossmann, 2021). This Research Topic was designed to allow editors from the section to highlight some of their own plant biotechnological work. There are many aspects of crops where this is needed - for example improving yields under changing climatic conditions will be crucial to help feed the growing world population - meaning that plant biotechnology is essential for food security. Plants are also good sources of pharmaceutically active compounds and can also be genetically manipulated to make them useful platforms for producing pharmaceutical proteins. Such plants where increased amounts of pharmaceuticals can be isolated, will help many medical treatments by reducing costs. Genetic manipulation of plants underlies much plant biotechnology and this can range from traditional plant breeding, through to transgenic and genome editing technologies. Understanding and improving the uses of these technologies will allow plant biotechnologists to improve plants in a more efficient manner. This Research Topic was designed to examine a wide range of different plant biotechnological issues, from understanding and overcoming abiotic stress tolerance, to manipulating specialized metabolism and the development of genome editing techniques.

One major aspect of plant biotechnology that will need much effort is to overcome different abiotic stresses as climate change is already affecting crop yields through increasing these types of stresses (Ray et al., 2019) and will likely only be worsened by future climate change. Khan et al. examined this by testing how wheat can grow better under salt stress when exposed to an endophytic fungus. They showed that this interaction leads to alterations in primary and secondary metabolites through hormonal regulation that help overcome saline stress. The work highlights the potential of including plant-endophyte interactions when considering biotechnological means to improve plant stress tolerance. Iron deficiency or uptake efficiency can also be a considerable limit on plant growth and resilience. Liu et al. identified a tobacco mutant that grows better with little iron. They characterized it further phenotypically and employed transcriptome analyses showing differences in gene expression related to molecular and physiological changes. RT-qPCR-based gene expression studies heavily depend on the availability of appropriate reference genes. Li et al. identified a set of reference genes in ginger, suitable for studying abiotic stress responses and postharvest biology studies. Finally, El-Sappah et al. reviewed various aspects of heat stress that affect maize production and suggest crop management and molecular breeding approaches to mitigate the effects.

Crop losses due to biotic stresses can be devastating and two papers were published examining these. In one study Bettoni et al. use *in vitro* techniques and combined chemical and thermal treatments to eliminate multiple viruses from potato plants to allow the production of virus-free plants. This procedure can play an important role in production of virus-free potato plants for farmers and can significantly improve the production of good quality potatoes. He et al. utilized genomics approaches to help in speed breeding an already high-yielding rice variety to incorporate resistance to two different bacterial diseases. This variety will be important for farmers through producing higher yields with reduced need for application of antibacterial chemicals. Although microbial infections are important, many crop losses are caused by weeds (Oerke, 2006) and Wong et al. provide a review on concepts how biotechnology (including gene drives) might serve weed management in the future.

Many compounds with pharmaceutical properties are found in plants. Further, understanding and manipulating specialized metabolism is a good way to identify and produce new drugs. A survey of orchid secondary metabolism was undertaken by Ghai et al. (2022) and utilized high throughput transcriptomics data to elucidate the role of potential candidates in secondary metabolite biosynthesis. Their findings may help in identifying interesting new compounds useful for drug development and the enzymes that synthesize them. Thorat et al. examined growth conditions that influenced accumulation of pharmaceutically important withanolides compounds in *Withania somnifera*. Using a transcriptomic approach they identified genes which are differentially expressed. Their findings could potentially be used to establish tools for rapid *in vitro* multiplication of *Withania* sp. and increase withanolides accumulation in this plant. *In vitro* cultivation and regeneration of plants is a key step for the biotechnological interventions in crops. Bull and Michelmore summarizes the current knowledge on molecular determinants of *de novo* organogenesis and somatic embryogenesis.

Plants can also be used to manufacture recombinant proteins (molecular farming), some of which can be used as pharmaceuticals or therapeutics. Such plant-based systems can have some advantages over production of the same proteins in microbes or mammalian cell cultures. One of these advantages is the easier production of heterologous glycoproteins. van der Kaaij et al. examined how this is advantageous to produce helminth glycoproteins with unusual glycan structures in plants, which can be used to treat autoimmune diseases. Two papers also reported on the production of active recombinant proteins in the forms of human transcriptional growth factor β1 (Soni et al.) or a bacterial laccase (van Eerde et al.). These proteins have pharmaceutical or biotechnological applications respectively and their production in plants should help reduce their cost.

Although modern biotechnological techniques, such as genome editing, offer novel and complementary options, traditional genetics are still crucial in improving crops. Coupled with genomic techniques this becomes increasingly important in identifying the genetic basis of traits and speed breeding these into elite varieties. Hu et al. describe the sequencing of three Chinese chestnut varieties and generation of a pangenome that will be incredibly useful for breeding efforts. Two other studies used genomic techniques: Wang et al. identify loci involved in wheat spike production through exome capture sequencing and RNAseq analysis. Similarly, Wen et al. performed a genome wide association study to improve biofortification by identifying genetic markers associated with lower phytate content in wheat grains. This will help increase availability of iron and zinc and improve the nutritional value of the grain. Finally, haplotype analysis of jujube chloroplasts was described, where they used information for 65 chloroplast genomes and will be useful for phylogenetic studies and breeding efforts in this plant (Hu et al.).

The importance of genome editing techniques in improving crop plants is broadly emphasized and a number of reviews on this subject were published. Dhugga highlighted that such techniques can speed up production of improved elite varieties with only 2-3 generations needed for variety development rather than the 5-6 that are currently required. Naik et al. examined potential interrelations between genome editing and nanotechnology for plant improvement, while Silva and Fontes examined how genome editing, especially using the CRISPR/Cas-system could help develop broad range viral resistance in plants. Despite the growing importance of genome editing for introducing mutations into plant genomes, post-transcriptional gene silencing can still play an important role in both gene function discovery and crop improvement. Imran et al. examined how nested secondary structure of miR159 influences silencing in *Arabidopsis thaliana*.

## Perspective

This Research Topic scratches the surface of several aspects of plant biotechnology ranging from applied demonstrations of biotechnological solutions to problems, to descriptions of new technologies that will become increasingly important. Plant biotechnology is a broad topic which overlaps many different scientific fields. We hope that this contributes to helping the plant science community in understanding some aspects of applied plant science, how they are currently used and how they will be utilized in the future by plant biotechnologists.

## Author contributions

JRL wrote the first draft of the manuscript and all authors contributed to, and approved the final version.
